# Tuberculosis infection among homeless persons and caregivers in a high-tuberculosis-prevalence area in Japan: a cross-sectional study

**DOI:** 10.1186/1471-2334-11-22

**Published:** 2011-01-21

**Authors:** Takahiro Tabuchi, Toshio Takatorige, Yukio Hirayama, Nobuaki Nakata, Shigeyoshi Harihara, Akira Shimouchi, Koshiro Fujita, Hiroko Yoshida, Yoshitaka Tamura, Takayuki Nagai, Tomoshige Matsumoto, Tetsuya Takashima, Hiroyasu Iso

**Affiliations:** 1 Public Health, Department of Social and Environmental Medicine, Graduate School of Medicine, Osaka University, 2-2 Yamadaoka, Suita-shi, Osaka, Japan; 2 Osaka Socio-Medical Center Hospital, 1-3-44 Haginochaya, Nishinari-ku, Osaka-shi, Osaka, Japan; 3 Osaka City Public Health Office, 1-2-7-1000, Asahimachi, Abeno-ku, Osaka-shi, Osaka, Japan; 4 Osaka Prefectural Hospital Organization Osaka Prefectural Medical Center for Respiratory and Allergic Diseases, 3-7-1 Habikino, Habikino-shi, Osaka, Japan

## Abstract

**Background:**

Tuberculosis (TB) is a major public health problem. The Airin district of Osaka City has a large population of homeless persons and caregivers and is estimated to be the largest TB-endemic area in the intermediate-prevalence country, Japan. However, there have been few studies of homeless persons and caregivers. The objective of this study is to detect active TB and to assess the prevalence and risk factors for latent TB infection among homeless persons and caregivers.

**Methods:**

We conducted a cross-sectional study for screening TB infection (active and latent TB infections) using questionnaire, chest X-ray (CXR), newly available assay for latent TB infection (QuantiFERON-TB Gold In-Tube; QFT) and clinical evaluation by physicians at the Osaka Socio-Medical Center Hospital between July 2007 and March 2008. Homeless persons and caregivers, aged 30-74 years old, who had not received CXR examination within one year, were recruited. As for risk factors of latent TB infection, the odds ratios (OR) and 95% confidence intervals (95% CI) for QFT-positivity were calculated using logistic regression model.

**Results:**

Complete responses were available from 436 individuals (263 homeless persons and 173 caregivers). Four active TB cases (1.5%) among homeless persons were found, while there were no cases among caregivers. Out of these four, three had positive QFT results. One hundred and thirty-three (50.6%) homeless persons and 42 (24.3%) caregivers had positive QFT results. In multivariate analysis, QFT-positivity was independently associated with a long time spent in the Airin district: ≥10 years versus <10 years for homeless (OR = 2.53; 95% CI, 1.39-4.61) and for caregivers (OR = 2.32; 95% CI, 1.05-5.13), and the past exposure to TB patients for caregivers (OR = 3.21; 95% CI, 1.30-7.91) but not for homeless persons (OR = 1.51; 95% CI, 0.71-3.21).

**Conclusions:**

Although no active TB was found for caregivers, one-quarter of them had latent TB infection. In addition to homeless persons, caregivers need examinations for latent TB infection as well as active TB and careful follow-up, especially when they have spent a long time in a TB-endemic area and/or have been exposed to TB patients.

## Background

Globally, there were an estimated 9.27 million cases of tuberculosis (TB) in 2007, with the larger number of cases of latent TB infection [1]. The Airin district of Osaka City is known as an urban area with a dense population of day-laborers and homeless people in Japan, an intermediate-prevalence country [2]. The prevalence of active TB in the Airin district was approximately 1,000 per 100,000 in the early 2000s, which was the highest in Japan and similar to that in developing countries [1,3,4]. In the Airin district, there are estimated to be 15,000 to 20,000 homeless persons with no medical insurance, accounting for 80% of visitors to a free or low-cost hospital, i.e. Osaka socio-medical center hospital [3]. TB screening programs have been carried out at Airin Health Office since 1990 and at the Osaka socio-medical center hospital since 2005. Mobile screening for TB was carried out monthly between 1973 and 2005, and weekly since 2006, by the Osaka City government on the streets of the Airin district [5]. The directly observed therapy short course (DOTS) program for homeless persons has been carried out since 1999. To date, several communities (i.e., NGOs, hospitals and facilities) for accommodation, health and welfare have been organized for homeless persons, following the Stop TB Strategy [5,6]. The prevalence of active TB in Osaka City decreased by 50% from 2000 to 2008. However, there are still estimated to be many unknown active TB cases in the Airin district [5].

Previous studies have identified risk factors for TB, such as immigrants, HIV, poverty, incarceration, smoking and alcohol use [7-10]. Homelessness-related TB remains a widespread problem [7]. An anti-TB strategy targeting homeless people, empowering caregivers and communities, and promoting research has been recommended [6,7]. However, studies on TB infection of homeless persons and their caregivers are scarce [11-13].

The commercially available blood test, QuantiFERON-TB Gold In-Tube (QFT; Cellestis Limited, Carnegie, Australia), is an interferon-gamma release assay (IGRA) in response to *M. tuberculosis*-specific antigens [14], and has been validated [15]. The QFT has excellent specificity and gives us valuable information of latent TB infection, even for a Japanese Bacillus Calmette-Guérin (BCG)-vaccinated population, whereas the accuracy of the tuberculin skin test (TST) is hampered by poor specificity due to the widespread use of BCG vaccination and re-vaccination in Japan [16].

The principal mechanism for the Stop TB Strategy is the detection and treatment of patients with TB [1]. The objectives of this study were to detect active TB cases and to assess the prevalence and risk factors of latent TB infection among homeless persons and their caregivers in a TB-endemic area in an intermediate-prevalence country.

## Methods

### Participants and Measurements

We conducted a cross-sectional study of homeless persons and their caregivers in the Airin district. Homeless persons were defined as persons who had had no permanent residence for more than one month. Caregivers were defined as persons who worked and supported homeless persons with regard to job arrangement, medical care, food supply, accommodation and clothing in the Airin district. Caregivers who belonged to five NGOs (mainly associated with a homeless shelter, job assistance, anti-alcoholism action, a soup-run and DOTS), as well as staff at the Osaka socio-medical center hospital and two clinics (nurses, social workers, dietitians, counselors and physicians) were enrolled in this study. Through our network of five NGOs, Osaka socio-medical center hospital and two local clinics by the use of posters, handouts and personal communication through a study recruiter, homeless persons and caregivers aged 30-74 years who had not received chest X-ray (CXR) examination within one year were recruited for tuberculosis screening at Osaka socio-medical center hospital between July 2007 and March 2008.

CXR, QFT, questionnaire and clinical evaluation by physicians were performed. If a participant had the symptom of sputum, smear testing and culture of sputum were performed for active TB diagnosis. A physician's interview using a standardized questionnaire covered the following data: age, sex, past history of TB, use of immunosuppressive drugs, past exposure to patients diagnosed with tuberculosis, current smoking and drinking status (more than 10 g of ethanol almost every day, yes or no), present symptoms of cough and/or sputum, general fatigue, elevated body temperature (≥37.0°C) and years spent living and/or working in the Airin district. Past exposure to tuberculosis patients was defined as self-reported exposure through living and/or working with TB patients in a shared space before the patients had been diagnosed with active TB.

CXR findings were categorized into two groups, normal and abnormal, based on our standard method [4]. Further classification was not performed since TB shadows vary and could take any kind of shape [17]. Quality control was performed by double-checks on each radiograph by another TB specialist. Active TB case was defined as an individual with symptoms compatible with TB plus detection of nucleic acid from mycobacterium tuberculosis complex from a clinical specimen, or as a patient with tuberculosis clinically suspected by an expert physician plus a response to anti-tuberculosis treatment [18].

QFT was performed and interpreted according to the manufacturer's instructions, with an interferon-γ response for the tuberculosis antigen tube minus Nil of ≥0.35 IU/ml defined as a positive result. QFT results were considered indeterminate if the subject did not respond to the mitogen-positive control tube with at least 0.5 IU/ml of interferon-γ [19,20].

Written informed consent was provided by each participant and each received 500 yen (about five US dollars) as an incentive to participate. The ethical committees of Osaka socio-medical center hospital and Osaka University approved this study.

### Statistical Analysis

The proportions of basic characteristics and TB-related findings were descriptively shown among homeless persons and caregivers. The prevalence and 95% confidence intervals (95% CI) for active TB were calculated using method based on the F-distribution.

The odds ratios (OR) and 95% CI for QFT-positivity were calculated using logistic regression model. We assessed how the positive QFT prevalence varied according to the potential factors such as length of time spent living and/or working in the Airin district and past exposure to TB patients, using the dichotomized categories to avoid statistical instability derived from small sample size. We investigated the multivariate model among homeless persons and caregivers, who did not have active TB disease or indeterminate QFT result.

Because of the failure in convergence, we removed the variables that had <5% cases with exposure or non-exposure category from the age-adjusted and multivariate logistic model [21]. Thereby, female and elevated body temperature for homeless, cough and/or sputum, elevated body temperature, past history of TB and abnormal chest X-ray finding for caregivers were excluded from the analyses.

Probability values for statistical tests were two-tailed and *p *< 0.05 was regarded as statistically significant. The SAS statistical software package (version 9.1; SAS Institute Inc., Cary, NC, USA) was used for all analyses.

## Results

A total of 448 persons were enrolled in the study. However, of these 448 participants, four homeless persons refused to give a blood sample, and eight homeless persons left the waiting room before having a CXR and/or completing a questionnaire. Thus, complete results of CXR, QFT and questionnaire were available for 436 participants (263 homeless persons and 173 caregivers).

Table 1 shows the basic characteristics and TB-related findings for homeless persons and caregivers. The homeless persons included only two women and exhibited higher values for almost all variables of interest than caregivers. Four active TB cases were found among the homeless persons: the prevalence of 1.52% (95% CI, 0.42-3.85), while there were no cases among caregivers. One-half of the homeless persons (50.6%) and one-quarter of the caregivers (24.3%) had a positive QFT result.

**Table 1:** Basic characteristics and TB-related findings of the study participants.

		Homeless persons (n = 263)	Caregivers (n = 173)
Basic characteristics			
Sex, female	n (%)	2 (0.8)	94 (54.3)
Age, years	mean median (range)	58.0 59 (30-74)	48.2 48 (30-71)
30-49	n (%)	40 (15.2)	97 (56.1)
50-59	n (%)	108 (41.1)	52 (30.1)
60-74	n (%)	115 (43.7)	24 (13.9)
Current smoker	n (%)	189 (73.0)	63 (37.1)
Current drinker	n (%)	134 (51.9)	54 (32.0)
Length of time spent living and/or working in the Airin district, years	mean median (range)	13.5 10 (0.1-58)	10.3 5 (0.1-64)
<5 years	n (%)	63 (25.9)	73 (43.2)
5-10 years	n (%)	46 (18.9)	33 (19.5)
≥10 years	n (%)	134 (55.1)	63 (37.3)

TB-related findings			
Past history of TB	n (%)	33 (12.6)	6 (3.5)
Past exposure to TB patients	n (%)	50 (19.8)	32 (18.7)
Cough and/or sputum	n (%)	64 (24.6)	6 (3.5)
General fatigue	n (%)	51 (19.6)	9 (5.2)
Elevated body temperature	n (%)	9 (3.5)	1 (0.6)
Abnormal chest X-ray finding	n (%)	68 (25.9)	7 (4.1)
Active TB case	n (%)	4 (1.5)	0 (0.0)
Positive QFT result	n (%)	133 (50.6)	42 (24.3)
Indeterminate QFT result	n (%)	4 (1.5)	1 (0.6)

Abbreviations: TB, tuberculosis; QFT, QuantiFERON-TB Gold In-Tube

The number of missing values was 20 for length of time spent in the Airin district, 10 for past exposure to TB patients, 4 for smoking, 5 for drinking and 3 for cough and/or sputum, general fatigue and elevated body temperature among homeless persons, and 4 for length of time spent in the Airin district, 2 for past exposure to TB patients, 3 for smoking and 4 for drinking among caregivers.

None of the participants had a history of extra-pulmonary TB or used immunosuppressive agents.

Table 2 shows the characteristics of active TB cases. Out of the four active TB cases, all four subjects were homeless, male, more than 60 years of age, had abnormal CXR findings, had lived or worked for more than 9 years (mean 13 years) in the Airin district and had symptoms of cough and/or sputum. Three subjects had positive QFT results. None of them had acknowledged past exposure to TB patients.

**Table 2:** Characteristics of active TB cases.

	Case 1	Case 2	Case 3	Case 4	Total*
Homeless or caregivers	Homeless	Homeless	Homeless	Homeless	4
Sex	Male	Male	Male	Male	4
Age	62	63	66	66	Mean 64.3
Current smoker	-	+	+	-	2
Current drinker	-	+	-	+	2
Length of time spent living and/or working in the Airin district, years	10	9	20	13	Mean 13.0
Past history of TB	-	+	-	-	1
Past exposure to TB patients	-	-	-	-	0
Cough and/or sputum	+	+	+	+	4
General fatigue	+	-	+	+	3
Elevated body temperature	-	-	-	-	0
Abnormal chest X-ray finding	+	+	+	+	4
QFT-positivity	-	+	+	+	3

Abbreviations: TB, tuberculosis; QFT, QuantiFERON-TB Gold In-Tube

*The number of positive for each factor or mean value for continuous variables among four active TB cases.

Table 3 shows age-adjusted and multivariate OR (95% CI) of QFT-positivity according to potential risk factors among homeless persons and caregivers, who did not have active TB disease or indeterminate QFT result. Both homeless persons and caregivers who had spent more than ten years in the Airin district had significantly higher positive QFT result than those who had spent less than ten years in both age-adjusted and multivariate models. When we used a cutoff point of half of the period, namely, five years, homeless persons who had spent more than five years had significantly higher positive QFT result (multivariate odds ratio, 2.93; 95% CI, 1.43-6.01) but caregivers did not (multivariate odds ratio, 1.93; 95% CI, 0.84-4.45). Current drinker was a significant predictive factor for QFT-positivity among homeless persons, but not among caregivers in both age-adjusted and multivariate models. The caregivers who had acknowledged past exposure to TB patients had significantly higher QFT-positivity than those who did not in both age-adjusted and multivariate models, while the homeless persons did not in the multivariate model.

**Table 3:** Age-adjusted and multivariate odds ratios (OR) and 95% confidence intervals (95% CI) of QFT-positivity according to potential risk factors among homeless persons and caregivers, after excluding the subjects with active TB disease or indeterminate QFT results.

		Homeless persons (n = 255)				Caregivers (n = 172)			
			
		No. of subjects	No.(%) of QFT-positivity	Age-adjusted OR (95% CI)	MultivariateOR (95% CI)†	No. of subjects	No.(%) of QFT-positivity	Age-adjusted OR (95% CI)	MultivariateOR (95% CI)†
Sex	Male	253	130 (51.4)	NA	NA	79	20 (25.3)	1.0	1.0
	Female	2	0 (0.0)	NA	NA	93	22 (23.7)	0.95 (0.46-1.93)	0.91 (0.39-2.11)
Current smoker	No	66	33 (50.0)	1.0	1.0	106	27 (25.5)	1.0	1.0
	Yes	185	93 (50.3)	1.19 (0.66-2.14)	1.23 (0.63-2.40)	63	13 (20.6)	0.81 (0.38-1.75)	0.72 (0.29-1.74)
Current drinker	No	121	56 (46.3)	1.0	1.0	114	26 (22.8)	1.0	1.0
	Yes	129	70 (54.3)	1.74 (1.02-2.96)*	1.84 (1.01-3.37)*	54	13 (24.1)	1.23 (0.56-2.69)	1.15 (0.48-2.74)
Length of time spent living and/or working in the Airin district, year	<10 years	107	38 (35.5)	1.0	1.0	105	18 (17.1)	1.0	1.0
	≥10 years	128	78 (60.9)	2.52 (1.46-4.35)*	2.53 (1.39-4.61)*	63	24 (38.1)	2.60 (1.24-5.42)*	2.32 (1.05-5.13)*
Past exposure to TB patients	No	196	96 (49.0)	1.0	1.0	138	27 (19.6)	1.0	1.0
	Yes	50	31 (62.0)	2.05 (1.05-3.99)*	1.51 (0.71-3.21)	32	14 (43.8)	3.63 (1.55-8.47)*	3.21 (1.30-7.91)*
Cough and/or sputum	No	194	100 (51.6)	1.0	1.0	166	42 (25.3)	NA	NA
	Yes	59	29 (49.2)	0.91 (0.50-1.67)	0.64 (0.32-1.32)	6	0 (0.0)	NA	NA
General fatigue	No	207	102 (49.3)	1.0	1.0	163	40 (24.5)	1.0	1.0
	Yes	46	27 (58.7)	1.66 (0.84-3.29)	1.47 (0.66-3.28)	9	2 (22.2)	0.96 (0.19-4.93)	1.06 (0.19-5.96)
Elevated body temperature	No	244	124 (50.8)	NA	NA	171	42 (24.6)	NA	NA
	Yes	9	5 (55.6)	NA	NA	1	0 (0.0)	NA	NA
Past history of TB	No	225	109 (48.4)	1.0	1.0	166	37 (22.3)	NA	NA
	Yes	30	21 (70.0)	2.23 (0.95-5.21)	1.51 (0.43-5.31)	6	5 (83.3)	NA	NA
Abnormal chest X-ray finding	No	193	86 (44.6)	1.0	1.0	165	37 (22.4)	NA	NA
	Yes	62	44 (71.0)	2.62 (1.39-4.93)*	1.90 (0.78-4.60)	7	5 (71.4)	NA	NA

*p < 0.05

Abbreviations: TB, tuberculosis; NA, not applicable

†Multivariate-adjusted for the listed factors and age.

Past history of TB and abnormal chest X-ray finding were not associated significantly with positive QFT result among homeless persons in the multivariate model, and the analysis was not carried out for caregivers owing to the small number with a past history or abnormal chest X-ray finding.

## Discussion

### Active TB prevalence

We showed that homeless people in the Airin district remain at high risk for active TB, and that the cross-sectional estimate of the prevalence of active TB was a rate of 1.52% (95% CI, 0.42-3.85) among homeless people in the Airin district, Osaka, Japan. A similar high prevalence of active TB was reported among homeless day-laborers (2.2%) in the same district from a previous study in 2003-2005 [4]. According to annual TB patients' registry database, the prevalence of active TB among residents in the Airin district was 653 per 100,000 (0.65%) in 2007, and the homeless people accounted for 79.3% of TB patients in that district [22]. These estimates of active TB prevalence were markedly higher than those of the total Japanese population and the Osaka City population in 2007, namely, 19 per 100,000 (0.019%) and 53 per 100,000 (0.053%), respectively [1,22].

High prevalence of active TB among the homeless population was reported from other countries. In New York City, McAdam et al. reported the high active TB prevalence of 1,502 per 100,000 among homeless persons in 1992 [23]. In London, Story et al. showed the high prevalence of 788 per 100,000 among homeless people in 2003 [24]. TB is concentrated in the homeless population in developed countries and TB transmission may occur from homeless people to other populations [25].

The trends for active TB prevalence among homeless people varied considerably by location. In New York City, the prevalence had a substantial decline, that is, 1,502 per 100,000 in 1992 to 171 per 100,000 in 2004 [23]. In the Airin district, where approximately 80% of residents were estimated to be homeless people, the prevalence declined substantially from 1400 per 100,000 in 2000 to 680 in 2005, and declined further to a small extent to 653 per 100,000 in 2007 [22].

The substantial decline in the active TB prevalence in New York City and the moderate decline in the Airin district among homeless people were attributable to intensive population-based TB screening and DOTS [5,26].

### Prevalence of latent TB infection

We found high prevalence of QFT-positivity for homeless persons (50.6%) and for caregivers (24.3%) at the ages of 30-74 years. When we restricted the sample to those of ages 40-69 years, the respective proportions were 50.4% for homeless persons and 30.8% for caregivers. These prevalences were far higher than that of the general Japanese population aged 40-69 years, which is estimated to be 7.1% [27].

### Latent TB infection among vulnerable persons including homeless people

Garfein et al. investigated latent TB infection among 280 homeless persons in a Mexican city with the highest TB prevalence using an IGRA and found the prevalence of QFT-positivity was 51.8% [28], which was nearly equal to the prevalence in our study. In addition to homeless people, high-risk individuals for latent TB infection can be detected by the IGRA. For example, the prevalence of IGRA-positivity was reported to be 29.8% among immigrants, mostly from Latin America, in Italy [29], 33.6% among drug users in Houston, USA [30], and 53.9% among immigrants with close contact to sputum smear-positive TB patients in Netherlands [31].

### Latent TB infection among caregivers

A study in Italy showed that the prevalence of latent TB infection (positive QFT) was 55.5% among caregivers working at a homeless shelter [11], which showed higher latent TB prevalence than the caregivers in our study. However, compared with healthcare workers for latent TB infection, the caregivers of our study had higher prevalence of latent TB infection. The prevalence of latent TB infection among healthcare workers in low- to intermediate-prevalence countries including Japan ranged from 1% to 19% [15,32-35], which was much lower than that of the caregivers in our study. Mirtskhulava et al. reported an extremely high prevalence of latent TB infection (60.0%) among healthcare workers, probably because they had frequent contact with TB patients and also high prevalence of TB in the community [36].

### Putative risk factors for latent TB infection

Another aim of our study was to determine putative risk factors for latent TB infection. QFT-positivity was associated with past exposure to TB patients among caregivers, but not among homeless people. Caregivers usually know when and how they have been in contact with people with TB, whereas homeless persons often ignored or did not notice this [37]. Homeless people who drank almost every day had higher QFT-positivity than those who did not. Habitual drinkers may be more likely to have contact with other drinkers and had a higher risk of being infected [38]. The past history of TB was not significantly associated with a positive QFT result among homeless persons, which might be related to the waning of immune responses in the time course of TB infection [27]. The duration of living and/or working in Airin district was associated with QFT-positivity among both homeless people and caregivers, but this was the case only for ≥ 5 years among homeless people.

The risk of QFT-positivity was found to increase with increasing length of time spent in the Airin district, independently of acknowledgement of exposure to TB patients. Even caregivers without known exposure to TB patients in this study had approximately twofold higher QFT-positivity (19.6%, Table 3) than healthcare workers in Japan (9.9%) [16], indicating that they may be at high risk for TB infection. As mentioned above, caregivers at a homeless shelter had high latent TB prevalence even in a low-prevalence country [11]. Thus, anti-TB measures for caregivers should be strengthened to ensure their safety.

## Limitations

This study has several potential limitations. The setting and selection of homeless persons in our study may limit the ability to generalize our results to the entire Airin district. Our sample of homeless persons may be at high risk to have been in contact with TB patients. The information on homelessness, past history of TB, past exposure to TB patients and length of time spent living and/or working in the Airin district was self-reported. Homeless people may be less likely to recall such information accurately because they are less health-conscious. The cross-sectional nature of the data limits the degree to which we can assign causality, especially with respect to temporality. However, it might be plausible that the exposure, such as past exposure to TB patients and length of time spent in the Airin district, may precede TB infection or disease. Information regarding TB-related factors such as HIV infection, drug abuse and history of incarceration was not included in the data collection. However, it is well known that TB/HIV co-infection is quite low in Japan [39]. In the present study, TST was not carried out because we wanted to avoid the refusal of study participation by homeless persons, and there is poor agreement between TST and QFT results caused by the effect of BCG vaccination in Japan [16].

## Conclusions

We found that the prevalence of latent TB infection was approximately 50% for homeless people and 25% for caregivers, and a long duration spent by both groups in the Airin district in Osaka, Japan, was associated with latent TB infection. Although no active TB was found for caregivers, one-quarter of them had latent TB infection. In addition to homeless persons, caregivers need examinations for latent TB infection as well as active TB and careful follow-up, especially when they have spent a long time in a high TB prevalence area and/or have been exposed to TB patients.

## Competing interests

The authors declare that they have no competing interests.

## Authors' contributions

T. Takatorige, YH, NN, SH, AS, KF, HY and T. Takashima participated in the planning of the study. T. Takatorige and T. Takashima coordinated the study and took overall responsibility for the delivery of the work. T. Tabuchi, YH, KF and HY had responsibility for data collection. T. Tabuchi conducted the analysis, with statistical support from HI, YT, TN and TM. T. Tabuchi, HI and T. Takashima participated in writing the paper. All authors participated in the interpretation of the study and read and approved the final manuscript.

## Pre-publication history

The pre-publication history for this paper can be accessed here:

http://www.biomedcentral.com/1471-2334/11/22/prepub
